# Eating cognitions, emotions and behaviour under treatment with second generation antipsychotics: A systematic review and meta-analysis

**DOI:** 10.1192/j.eurpsy.2023.237

**Published:** 2023-07-19

**Authors:** H. Mutwalli, J. L. Keeler, S. Bektas, N. Dhopatkar, J. Treasure, H. Himmerich

**Affiliations:** 1 Institute of Psychiatry, Psychology and Neurosciences, King’s College London, London, United Kingdom; 2 Department of Clinical Nutrition, Imam Abdulrahman Bin Faisal University, Dammam, Saudi Arabia; 3 Institute of Psychiatry, Psychology and Neuroscience, King’s College London; 4 Eating Disorders Unit, South London and Maudsley NHS Foundation Trust (SLaM), London, United Kingdom

## Abstract

**Introduction:**

Weight gain and metabolic disturbances are frequent in people treated with second generation antipsychotics (SGA).

**Objectives:**

We aimed to investigate the effect of SGAs on eating behaviors, cognitions and emotions, as a possible contributor to weight gain and metabolic disturbances.

**Methods:**

A systematic review and meta-analysis was conducted following the Preferred Reporting Items for Systematic reviews and Meta-Analyses (PRISMA) guidelines. Original articles measuring outcomes relating to eating cognitions, behaviours and emotions, during treatment with SGAs were included in this review. A total of 92 papers with 11,274 participants were included from three scientific databases (PubMed, Web of Science and PsycInfo). Results were synthesized descriptively except for the continuous data where meta-analyses were performed and for the binary data where odds ratios were calculated.

**Results:**

Hunger was increased in participants treated with SGAs with an odds ratio for appetite increase of 1.51 (95% CI [1.04, 1.97]; z=6.40; *p*<0.001)(see Figure 1.). Compared to controls, our results showed that craving for fat and carbohydrates are the highest among other craving subscales. There was a small increase in dietary disinhibition (SMD=0.40) and restrained eating (SMD=0.43) in participants treated with SGAs compared to controls and substantial heterogeneity across studies reporting these eating traits (See figure 2 and 3). There were few studies examining other eating-related outcomes such as food addiction, satiety, fullness, caloric intake and dietary quality and habits.

**Image:**

**Figure:**
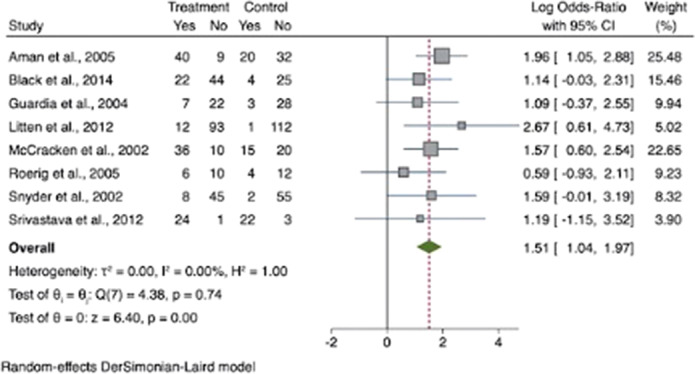

**Image 2:**

**Figure:**
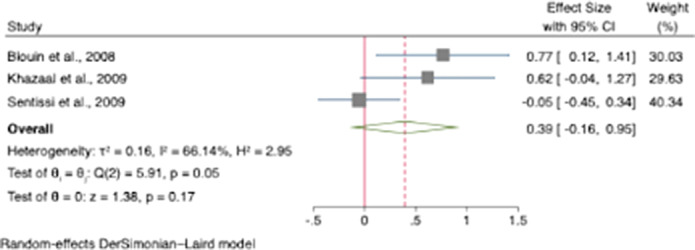

**Image 3:**

**Figure:**
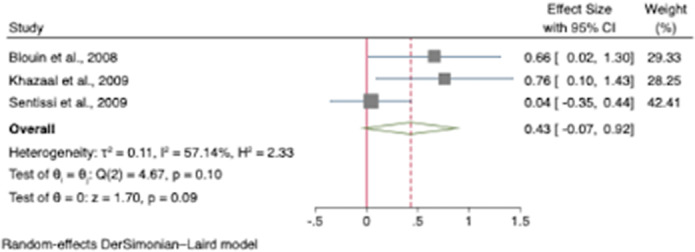

**Conclusions:**

Understanding the mechanisms associated with appetite and eating-related psychopathology changes in patients treated with antipsychotics is needed to reliably inform the development of effective preventative strategies.

**Disclosure of Interest:**

H. Mutwalli Grant / Research support from: The Saudi Arabian Government Educational Sponsorship for PhD, J. Keeler Grant / Research support from: The Medical Research Council, S. Bektas Grant / Research support from: The Turkish Ministry of National Education for PhD training, N. Dhopatkar Employee of: South London and Maudsley NHS Foundation Trust (SLaM), J. Treasure Grant / Research support from: The National Institute for Health Research (NIHR) Biomedical Research Centre (BRC) at the South London and Maudsley NHS Foundation Trust (SLaM), Employee of: King’s College London, H. Himmerich Grant / Research support from: The National Institute for Health Research (NIHR) Biomedical Research Centre (BRC) at the South London and Maudsley NHS Foundation Trust (SLaM), Employee of: King’s College London.

